# Accuracy of Herdsmen Reporting versus Serologic Testing for Estimating Foot-and-Mouth Disease Prevalence

**DOI:** 10.3201/eid2012.140931

**Published:** 2014-12

**Authors:** Kenton L. Morgan, Ian G. Handel, Vincent N. Tanya, Saidou M. Hamman, Charles Nfon, Ingrid E. Bergman, Viviana Malirat, Karl J. Sorensen, Barend M. de C. Bronsvoort

**Affiliations:** University of Liverpool, Neston, Wirral, UK (K.L. Morgan);; University of Edinburgh, Roslin, Scotland, UK (I.G. Handel, B.M. de C. Bronsvoort);; Institute of Agricultural Research for Development, Ngaoundéré, Cameroon (V.N. Tanya, S.M. Hamman, C. Nfon);; Ministry of Scientific Research and Innovation, Yaoundé, Cameroon (V.N. Tanya);; Pan American Foot and Mouth Disease Center, Rio de Janeiro, Brazil (I.E. Bergman, V. Malirat);; Danish Veterinary Institute for Virus Research, Kalvehave, Denmark (K.J. Sorensen);; Instituto de Ciencia y Tecnología Dr. César Milstein, Buenos Aires, Argentina (I.E. Bergman, V. Malirat)

**Keywords:** epidemiology, participatory, farmer reporting, validation, questionnaire, foot and mouth disease, veterinary, viruses, herdsmen

## Abstract

Herdsman-reported disease prevalence is widely used in veterinary epidemiologic studies, especially for diseases with visible external lesions; however, the accuracy of such reports is rarely validated. Thus, we used latent class analysis in a Bayesian framework to compare sensitivity and specificity of herdsman reporting with virus neutralization testing and use of 3 nonstructural protein ELISAs for estimates of foot-and-mouth disease (FMD) prevalence on the Adamawa plateau of Cameroon in 2000. Herdsman-reported estimates in this FMD-endemic area were comparable to those obtained from serologic testing. To harness to this cost-effective resource of monitoring emerging infectious diseases, we suggest that estimates of the sensitivity and specificity of herdsmen reporting should be done in parallel with serologic surveys of other animal diseases.

## Abstract 2

In an endemic area, herdsmen’s reports were as accurate as serologic test results.

Owner-, farmer-, or herdsman-reported disease prevalence is widely used in veterinary epidemiologic studies ( *1* – *6* ), especially for diseases that produce visible external lesions (e.g., ovine myiasis, foot-and-mouth disease [FMD]) ( *1* , *5* ) or characteristic clinical signs (e.g., scrapie) ( *7* ). For such interview- or questionnaire-based reporting, a common criticism is lack of external validation because questionnaires, like other measuring devices, need to be calibrated. External validation is usually approached by comparing questionnaire data with data measured by other methods such as visual inspection ( *8* – *10* ), photographs ( *11* ), selection of clinical signs ( *2* , *4* ), laboratory test results ( *12* ), or other ( *4* , *13* ). These approaches, however, are difficult to use in poorer countries and pastoral populations, where there are limited resources and no comparison data. We estimated sensitivity and specificity of herdsman-reported FMD prevalence in the Adamawa plateau, Cameroon, and compared herdsmen’s estimates with serologic test results.

FMD is a highly contagious viral disease of even-toed ungulates, caused by FMD viruses in the family *Picornaviridae* . Globally, FMD is a major disease of livestock because it leads to production losses and restrictions on trade with FMD-free countries ( *14* ). Clinical signs in cattle are distinct: vesicles on the tongue, gums, coronary band, and occasionally, udder. Animals salivate and are febrile, lame, and inappetant. Ruptured vesicles leave ulcers with characteristic underrun epithelial tissue at the edges ( *15* ).

To assess herdsmen’s ability to correctly identify FMD and to compare the sensitivity and specificity of herdsman reporting with that of serologic testing, we conducted a cross-sectional study of FMD on the Adamawa plateau, the major cattle-rearing area of Cameroon. We used a structured questionnaire, administered by interview, to determine whether herdsmen had seen FMD in their herds in the previous 1 and 2 years ( *5* *,* *16* ). Their ability to correctly identify FMD was also assessed by showing them color photographs of typical lesions. To estimate the sensitivity and specificity of the various estimates, we used Bayesian latent class models. These estimates were arrived at by restricting the age of cattle analyzed by virus neutralization (VN) testing to <2 years and by adopting evidence that nonstructural protein (NSP) antibody titers fall more rapidly (over ≈1 year) than VN antibodies ( *17* , *18* ). The study was conducted in accordance with the Cameroonian Ministry of Research guidelines and with approval from the University of Liverpool ethics committee in 1999. 

## Materials and Methods

### Study Population

The study population is described elsewhere ( *5* ). In brief, a database of 13,006 herds was constructed from rinderpest vaccination records from 88 veterinary centers across the Adamawa region. This region is ≈64,000 km^2^, lies between latitudes 6°N and 8°N, and is divided into 5 administrative divisions (Vina, Mbere, Mayo Banyo, Djerem, and Faro and Deo).

### Study Design

We used a cross-sectional study design and 2-stage stratified random cluster sample to select 147 herds in 2000. The sample size was chosen to enable a herd seroprevalence of 50% to be estimated with 9% accuracy and 90% confidence; we increased the number of samples selected by 10% (inflation) to allow for refusals ( *5* ).

From each herd, a minimum of 5 adult (>24 months) and 5 juvenile (8–24 months) cattle were randomly sampled ( *5* , *16* ). We used samples from juvenile cattle only. With a sample of this size, the probability of detecting at least 1 seropositive animal in a herd of 70 was 95%, assuming within-herd seroprevalence of 50% and test sensitivity and specificity of 100% each. The lower age limit was set at 8 months to minimize misclassification associated with maternal antibodies. In herds with <5 animals in the appropriate age group, all animals in that group were sampled. The number of animals presented for sampling from each herd was 7–81 (median 35, mean 37.4).

### Sampling

Blood was collected by jugular venipuncture into 10-mL Vacutainer tubes (Becton Dickinson, Franklin Lakes, NJ, USA), allowed to clot, and then separated in a 12-volt portable field centrifuge (Vulcon Technologies, Grandview, MO, USA). Serum was collected into two 1.8-mL cryovials (Nunc. Roskilde, Denmark) and kept at 4°C in a portable gas refrigerator for up to 14 days before being frozen and stored at –20°C. Samples were transported on dry ice to the World Reference Laboratory for Foot-and-Mouth Disease in Pirbright, UK, and stored at –20°C.

### Questionnaire

To collect data from herdsmen, we used a structured, interview-based questionnaire, administered in Fulfulde (the language of the Fulani people) ( *5* , *19* ). The questionnaire asked whether respondents had observed FMD in their herd in the previous year and (separate question) in the previous 2 years.

### Photographs

Herdsmen were asked to identify the diseases shown in 3 A4-sized photographs: a bovine tongue with a ruptured FMD vesicle, a bovine foot with ruptured FMD vesicles, and a bovid with lumpy skin disease ( *Capripoxviridae* , Poxviridae). The interviewer oriented the viewer as to what was on the photograph, pointing out relevant anatomic, but not pathologic, features. A herdsman was described as being able to recognize FMD if he identified at least 1 of the FMD photographs correctly and either identified or recognized lumpy skin disease as not being FMD.

### VN Testing

VN testing was performed according to the World Organisation for Animal Health/World Reference Laboratory protocol ( *20* ). Details are described elsewhere ( *21* ). VN results for each herd were then combined so that if positive results were found for any of the 3 virus serotypes (O, A, SAT2), that animal was considered positive.

### ELISA Testing

To test for antibodies against NSP, we used 3 ELISAs: indirect (I)–ELISA, CHEKIT-ELISA, and competitive (C)–ELISA. Each is described below.

For screening with the I-ELISA 3ABC (I-ELISA), aliquots of heat-treated serum (56°C for 2 h) were sent to Panaftosa, Brazil. This test is described elsewhere ( *22* , *23* ). Two samples had insufficient serum for the I-ELISA, so this testing was performed for 1,375 animals, 651 of which were 8–24 months of age.

The CHEKIT-3ABC-FMD ELISA (CHEKIT-ELISA) is described elsewhere ( *23* ). Testing was performed by author B.M.de C.B. at the World Reference Laboratory for Foot-and-Mouth Disease, according to the manufacturer’s instructions.

The C-ELISA was performed as described ( *24* , *25* ). Testing was conducted by author K.J.S. at the Danish Institute for Food and Veterinary Research in Kalvehave, Denmark.

### Comparison of Herdsman Reporting and Serologic Testing

First, herdsmen’s reports of disease in their herd in the previous 2 years were compared with VN test results for cattle 8–24 months of age in the same herd. Second, herdsmen’s reports of disease in the previous year were compared with antibodies against NSP determined by all 3 NSP ELISAs.

### Statistical Analyses

Prevalence estimates were conducted by using STATA version 6.0 ( http://www.stata.com ). To avoid bias in point and variance estimates, we incorporated stratification and cluster effects with *svymean* or *svyprop* commands and *strata* (administrative division), *psu* (veterinary center), and *pweight* (probability weightings) ( *5* ).

Sensitivity and specificity of serologic testing and herdsmen reporting were estimated by using a Bayesian latent class model ( *24* , *26* , *27* ) and the JAGS (Just Another Gibbs Sampler) ( http://mcmc-jags.sourceforge.net/ ) software package in R. This technique requires use of at least 2 tests that are conditionally independent (i.e., that if the true disease status of an animal were known, the outcome of 1 test would not influence the probability of a positive or negative result in the other). This technique also requires that prior distributions are specified for test properties and prevalence. The serologic tests were assigned a prior distribution of β (3,1) according to previous estimates of sensitivity and specificity ( *23* ). Herdsmen’s reports were assigned an uninformed distribution of β (1,1), which is equivalent to a uniform distribution between 0 and 1 and implies no prior knowledge of test performance.

Sensitivity and specificity were estimated by using a Markov chain Monte Carlo technique and Gibbs sampling ( *28* *,* *29* ), which involves sampling from the posterior distribution of interest and calculating the relevant measures (e.g., means, medians, and standard deviations of the parameters). This iterative procedure involves burn-in, checking for convergence of the sample chain, and then sampling from the posterior distribution. In this model, the first 50,000 iterations were discarded as burn-in, and every 100th of the following 200,000 iterations were kept for posterior inference. Convergence was assessed by visual inspection of the time-series plots for the parameters and by using Gelman and Rubin diagnostic plots from 3 sample chains with different starting values ( *30* ).

The posterior means, medians, and 95% credibility intervals (PCIs) for sensitivity, specificity, and prevalence were calculated. Because no differences between means and medians were found, means were reported; the primary results were 95% PCIs.

When comparing herdsmen’s reports of FMD in the previous 2 years with VN test results, sensitivity and specificity could not be allowed to vary across populations because there were only 2 tests. However when 3 NSP tests were used, sensitivity and specificity of herdsmen’s reports were allowed to vary across populations, depending on factors such as whether the herdsmen watched the animals daily, whether the owner was of Fulani or Mbororo ethnicity, or whether the herdsmen could recognize FMD lesions from pictures. To examine differences between prevalence and disease recognition in photographs, we used χ^2 ^testing.

.

## Results

### Response Rate

Of the 147 herds selected, 146 (99.3%) were sampled. Flooding prevented access to 1 herd. Blood was collected from 1,377 animals, 651 of which were 8–24 months of age (142 herds). One herd was excluded because antibody test results were missing, leaving 141 herds from which blood was collected.

### FMD Prevalence during Previous 2 Years

Herdsmen reported that 78.2% herds had been infected with FMD at least once during the previous 2 years. VN testing results indicated an estimated 80.3% prevalence ( Table 1 ). Prevalence estimated by both methods differed among administrative divisions. FMD in the previous 2 years was reported by all herdsmen in Faro and Deo but by only 55% in Djerem. Prevalence estimates obtained by VN testing were similar ( Table 1 ).

**Table 1:** Prevalence of FMD among cattle, Adamawa plateau, Cameroon, according to different surveillance methods, 2000*

Administrative division	Previous 2 years, % (95% CI)†			Previous 1 year, % (95% CI)†			
	Herdsmen’s reports	VN testing		Herdsmen’s reports	I-ELISA	CHEKIT-ELISA	C-ELISA
Vina	89.6 (83.0–96.1)	85.1 (76.4–93.8)		76.6 (66.4–86.8)	74.5 (64.9–84.0)	29.8 (16.3–43.2)	70.2 (58.6–81.8)
Mbere	72.0 (55.0–89.0)	76.0 (57.0–95.0)		54.4 (32.6–76.1)	50.8 (33.0–68.8)	15.8 (1.8–29.7)	56.1 (32.6–79.7)
Djerem	54.8 (34.7–74.9)	59.2 (48.0–70.6)		35.7 (19.1–52.3)	55.4 (45.0–65.7)	16.1 (1.7–30.4)	37.5 (24.1–50.9)
Mayo Banyo	78.6 (66.0–91.2)	85.7 (76.2–95.2)		43.9 (22.5–65.4)	59.1 (39.4–78.8)	12.1 (0.7–23.5)	63.6 (44.5–82.8)
Faro and Deo	100	100		73.3 (62.2–84.5)	73.3 (52.4–94.3)	40.0 (28.8–51.2)	73.3 (52.4–94.2)
Overall	78.2 (72.1–84.3)	80.3 (75.0–85.6)		57.4 (49.8–65.1)	63.0 (56.2–69.9)	21.8 (15.6–28.0)	60.4 (52.6–68.2)

*FMD, foot-and-mouth disease; VN, virus neutralization. †CIs adjusted for stratification by administrative division and clustering of herds by veterinary center.

### FMD Prevalence during Previous Year

For the previous year, ≈60% of herdsmen reported having noticed FMD in their herds. This prevalence estimate was similar to that obtained by I-ELISA and C-ELISA but considerably more than that estimated by CHEKIT-ELISA ( Table 1 ). The differences in reported prevalence among administrative divisions for the previous 2 years were also found for the previous year. ( Table 1 )

### Sensitivity and Specificity 

Overall sensitivity of herdsmen’s reports of FMD in the past 2 years was 95.7% (95% PCI 88.7%–99.8%) and specificity was 60% (95% PCI 44.3%–77.5%). These rates were remarkably similar to those determined by VN testing for serum antibodies in juvenile cattle (sensitivity 95.2% [95% PCI 89.6%–99.1%] and specificity 59.9% [95% PCI 45.6%–77.2%]).

Overall sensitivity of herdsmen’s reports of FMD in the previous year was 84.0% (95% PCI 75.1%–92.2%) and specificity was 75.1% (95% PCI 62.7%–85.1%). Sensitivity of herdsmen’s reports was significantly lower than that of I-ELISA (97.1% [95% PCI 91.0%–99.9%]) and C-ELISA (97.5% [95% PCI 91.9%–99.9%]). Specificity of herdsmen's reports was also slightly lower than that of I-ELISA (79.6% [95% PCI 68.0%–89.6%]) and C-ELISA (86.5% [95% PCI 75.1%–95.7%]) but not significantly so. Sensitivity was poor for CHECKIT-ELISA (37.2% [95% PCI 27.0%–48.1%]), but specificity was high (92.8% [95% PCI 85.0%–98.1%]).

Differences among administrative divisions were marked. The sensitivity of herdsmen’s reports was highest for Vina (94.3%) and lowest for Djerem (57.8%); specificity was highest for Mayo Banyo (92.0%) and lowest for Faro and Deo (33.1%) ( Table 2 .)

**Table 2:** No–gold standard estimation of herd-level sensitivity and specificity of herdsman reporting of FMD in administrative divisions of the Adamawa plateau, Cameroon*

Administrative division	Sensitivity, % (95% PCI)	Specificity, % (95% PCI)
Vina	94.3 (84.2–99.4)	70.6 (44.6–91.3)
Mbere	77.2 (50.7–96.5)	69.3 (42.0–91.0)
Djerem	57.8 (29.0–84.6)	73.1 (51.4–90.3)
Mayo Banyo	76.3 (52.8–95.0)	92.0 (72.8–99.8)
Faro and Deo	69.1 (42.9–90.4)	33.1 (5.2–71.4)
Overall	84.0 (75.1–92.2)	74.6 (62.7–85.1)

*FMD, foot-and-mouth disease; PCI, posterior credibility interval.

Sensitivity, but not specificity, of herdsmen’s reports differed among ethnic groups. Sensitivity was greater for the Fulani (90.3% [795% PCI 8.7%–98.0%]) than for the Mbororo people (73.8% [95% PCI 57.5%–87.5%]); p < 0.001. Specificity for the Fulani was 72.4% (95% PCI 53.2%–88.2%) and for the Mbororo was 76.4% (95% PCI 60.4%–89.5%). 

Reporting accuracy did not differ between herd owners and nonowners. Sensitivities were 79.3% (95% PCI 61.2%–92.8) and 82.9 (95% PCI 71.8%–92.2%), and specificities were 73.7% (95% PCI 52.0%–91.5%) and 74.4% (95% PCI 59.8%–86.8%), respectively. 

Similarly, reporting accuracy did not differ between respondents who watched cattle daily and those who did not. Sensitivities were 88.5% (95% PCI 75.6%–97.3%) and 76.9% (95% PCI 63.9%–88.2%), and specificities were 71.9% (95% PCI 49.6%–89.7%) and 75.1% (95% PCI 60.6–87.5%), respectively.

### Herdsmen Identification of FMD in Photographs 

FMD was correctly identified on 1 of 2 photographs by more than two thirds (69.3% [95% CI 61.4%–77.2%]) of herdsmen; 60.4% (95% CI 53.2%–67.7%) correctly identified FMD tongue lesions, 65.2% (95% CI 57.6%–72.8%) FMD foot lesions, and 55.8% (95% CI 47.8%–63.8%) both. Only 20.9% (95% CI 12.9%–28.8%) correctly identified FMD lesions in all 3 photographs. Lumpy skin disease was recognized by 28.5% (95% CI 19.9. Almost a quarter (24.3% [95% CI 17.1%–31.6%]) were unable to recognize FMD or lumpy skin disease from photographs.

Herd ownership did not influence ability to recognize FMD from photographs. FMD was recognized in photographs by 68.5% (95% CI 60.0%–76.9%) of owners and 71.3% (95% CI 58.5%–84.1%) of nonowners (p = 0.675).

Ethnicity affected the ability to recognize FMD from photographs. FMD lesions were recognized by a greater proportion of Fulani (82.2% [95% CI 72.3%–92.3%]) than Mbororo (58.8 % [95% CI 44.1%–73.6%]) herdsmen; p = 0.0143. 

Frequency of herd observation did not influence ability to recognize FMD from photographs. FMD lesions were recognized by 66.1% (95% CI 51.9%–80.2%) of those who watched the animals daily and by 70.7% (95% CI 61.2%–79.3%) of those who did not (p = 0.537).

Administrative region did affect ability to recognize FMD from photographs. Recognition of FMD lesions in photographs was highest for herdsmen in Vina (79.2% [95% CI 67.1%–91.2%]) and lowest for those in Faro and Deo (53.3% [95% CI 19.2%–87.5%]); these differences were not statistically significant (p = 0.354). FMD lesion recognition was 72.3% (95% CI 57.4%–89.3%) for herdsmen in Mbere, 59.4% (95% CI 38.9%–79.8%) in Djerem, and 68.2% (95% CI 51.6%–84.8%) in Mayo Banyo.

### Sensitivity and Specificity of Photograph Identification 

Compared with sensitivity for NSP antibody testing, sensitivity was higher for herdsmen recognition of FMD lesions in 1 photograph but specificity was lower for reporting of FMD in the previous year. The sensitivities and specificities were 90.0% (95% PCI 80.4%–97.3%) and 69.5% (95% PCI 54.3%–83.4%) for those able to identify a photograph of FMD compared with 63.5% (95% PCI 44.0%–90.9%) and 83.2% (95% PCI 64.0%–96.0%) for those who could not.

## Discussion

With regard to estimating herd prevalence of FMD, herdsmen performed as well as laboratory-based VN testing. Estimates of prevalence in the previous 2 years were 78.2% (95% CI 72.1%–84.3%) according to herdsman’s reports and 80.3% (95% CI 75.0%–85.6%) according to VN test results. Sensitivities of estimates for prevalence in the previous 2 years were 95.7% (95% PCI 88.7%–99.8%) and 95.2% (95% PCI 89.6%–99.1%) and specificities were 60.0% (95% PCI 44.3%–77.5%) and 59.9% (95% PCI 45.6%–77.2%), for herdsmen’s reports and VN test results, respectively. These estimates were derived by restricting the age of cattle to <2 years and by using a no–gold standard Bayesian model (model to assess diagnostic test performance in the absence of a perfect reference test) to estimate sensitivity and specificity.

In addition to validating estimates of FMD prevalence in the previous 2 years, we also attempted to validate farmer reporting for the previous year by taking a different approach. The rationale behind using tests that detect antibodies against NSP was that the number of animals <1 year of age in the sample was insufficient to produce generalizable results and that NSP antibody titers fall more rapidly over time than do VN antibody titers ( *17* , *18* ). In an evaluation study in which we reported that the CHECKIT-ELISA performed less well than the I-ELISA and C-ELISA, we used 3 NSP ELISAs ( *23* , *25* ). The results of the CHECKIT-ELISA are included in the study reported here because they enable comparison with results in the only other publication in which herdsmen’s estimates of FMD are compared with serologically derived estimates ( *12* ).

The 84.0% sensitivity of herdsmen’s reports of FMD in the previous year was significantly lower than the sensitivity of I-ELISA (97.1%) and the C-ELISA (97.5%) results. The 75.1% specificity of herdsmen’s reports was within the Bayesian credibility limits of the NSP test results. There are no published population-based estimates of NSP antibody persistence. In experimental studies, NSP antibodies have been detected in cattle for 229 ( *31* ), 304 ( *32* ), 365 ( *33* ), 395 ( *24* ), and 560 ( *17* ) days after infection, at which point the studies were terminated. It is possible that persistence of NSP antibody for >1 year accounted for the significantly lower sensitivity of herdsmen’s reports compared with serum antibodies against NSP (i.e., NSP serum antibodies represented infection over the previous 2 years, but herdsmen reporting was confined to 1 year, when fewer herds would have been seropositive). However, the lower seroprevalence according to VN testing (80.3%) compared with NSP ELISA seroprevalence (60.0%–64.5%) would argue against this.

The only test previously used to validate herdsmen’s reports of FMD is the CHEKIT-ELISA ( *12* ). When we used the results of this test as a reference standard, estimates of the sensitivity of reporting by pastoral Masai and Sukuma herdsmen in Tanzania were similar to those for herdsmen in Cameroon. Overall sensitivities were 90.9% (95% CI 75.7%–98.1%) and 72.7% (95% CI 49.8%–89.3%), respectively; however, specificities were lower at 35.2% (95% CI 14.2%–61.7%) and 35.1% (95% CI 20.2%–52.5%), respectively ( *13* ). The results of this and another study ( *19* ) suggest that the CHECKIT-ELISA was not the best choice of reference standard and that herdsmen’s estimates are more reliable.

By restricting the age of cattle to 8–24 months, we focused on recent herd exposure. The lower limit was chosen to avoid misclassification associated with presence of maternal antibodies. The upper limit means that herds infected during the last 2 weeks of the 2-year period might not have had time to seroconvert, but given a random distribution of infection in these herds over the 24-month period, only 2% (2/104weeks) of herds would have been infected during these last 2 weeks. 

In recent years, use of latent class models to estimate sensitivity and specificity of multiple tests in the absence of a reference standard has become common practice ( *34* ). A critical assumption of this technique is that test results must be independent within 2 classes ( *35* , *36* ), especially when a 2-class latent model is used. We used 2 biologically different and independent test approaches: herdsman reporting and VN testing. The assumption of conditional independence can be relaxed when there are >2 classes, but in our study, it was preserved even when 4 classes were compared; herdsmen reporting differed biologically from NSP ELISAs. A Bayesian approach to latent class models requires specification of prior distributions. The β (3,1) prior distributions given to NSP tests were based on previous findings. The uninformed β (1,1) prior distribution given to herdsman reporting is recommended when using this technique. Model fit was assessed by using Gelman-Rubin plots and statistics.

This study covered 64,000 km^2^ and 5 administrative divisions. Differences in reports of FMD prevalence were found for herdsmen ethnic groups, ownership status, and amount of cattle contact. However, the only variable for which a statistically significant difference was found was ethnic group; sensitivity of reporting by Fulani herdsmen was greater than that by Mbororo herdsmen. The Fulani and Mbororo are the major pastoralist groups on the Adamawa. They have a common language and cultural heritage, but the Mbororo are largely nomadic whereas the Fulani tend to be sedentary ( *37* ). The greater sensitivity of reporting by Fulani herdsmen is perhaps surprising because the nomadic group might be expected have more cattle contact. However, watching cattle on a daily basis was not associated with increased reporting accuracy. The differences between the Fulani and Mbororo might be a chance finding, or it might reflect differences in education or cattle ownership. A transethnic class of livestock owner seems to be emerging, in which sedentary Fulani employ non-Fulani herders, and non-Fulani owners employ poorer Mbororo who have lost their own herds. However, in this study, ownership was not associated with increased reporting accuracy.

With regard to the higher proportion of Fulani than Mbororo herdsmen who were able to identify FMD lesions from photographs, it is possible that Mbororo herdsmen might have less access to education and less experience interpreting 2-dimensional images ( *38* ). It is also possible that herdsmen rarely see vesicles in the mouth or coronary band and are more familiar with salivation and lameness. Recognition of lameness would be similar for sedentary and nomadic herdsmen because both groups spend each day slowly walking their cattle over a grazing area.

The finding of higher specificity for herdsmen recognition of FMD in at least 1 photograph and lower sensitivity of FMD reporting indicates a higher probability of reporting true-negative herds and a lower probability of reporting true-positive herds. This finding might represent a systematic reporting bias associated with herdsmen concerns about admitting that they had had FMD in their herds or a chance finding associated with seeing a familiar concept (FMD) in an unfamiliar way (photograph).

These results suggest that in FMD-endemic areas, an effective FMD surveillance method might be simply asking herdsmen if they have seen FMD in their herds. This concept is intuitive because FMD is a common disease and herdsmen are familiar with it. Whether herdsmen’s reports of FMD prevalence would be effective in countries where FMD is sporadic or less prevalent remains to be determined.

If our findings are generalizable to other diseases that produce visible clinical signs in other populations, herdsmen’s reports would provide a cost-effective surveillance mechanism that could extend to emerging diseases. In initial discussions, herdsmen reported that “Njobo” (Fulfulde word for FMD) had changed in recent years by causing death among adult cattle rather than just calves. The subsequent isolation of FMD virus serotype SAT2 in Cameroon provided a scientific explanation for this observation. Because of the potential usefulness of herdsmen’s observations in surveillance and emerging disease identification, we suggest that studies of animal disease prevalence in developing countries should include estimates of sensitivity and specificity of reporting. 
